# What Is the Optimal Duration of Adjuvant Mitotane Therapy in Adrenocortical Carcinoma? An Unanswered Question

**DOI:** 10.3390/jpm11040269

**Published:** 2021-04-04

**Authors:** Vittoria Basile, Soraya Puglisi, Barbara Altieri, Letizia Canu, Rossella Libè, Filippo Ceccato, Felix Beuschlein, Marcus Quinkler, Anna Calabrese, Paola Perotti, Paola Berchialla, Ulrich Dischinger, Felix Megerle, Eric Baudin, Isabelle Bourdeau, André Lacroix, Paola Loli, Alfredo Berruti, Darko Kastelan, Harm R. Haak, Martin Fassnacht, Massimo Terzolo

**Affiliations:** 1 Internal Medicine, Department of Clinical and Biological Sciences, San Luigi Gonzaga Hospital, University of Turin, 10043 Turin, Italy; basile_vittoria@libero.it (V.B.); anna.calabrese678@gmail.com (A.C.); oncotrial.sanluigi@gmail.com (P.P.); massimo.terzolo@unito.it (M.T.); 2 Division of Endocrinology and Diabetes, Department of Internal Medicine, University Hospital, University of Würzburg, 97080 Würzburg, Germany; altieri_b@ukw.de (B.A.); dischinger_u@ukw.de (U.D.); megerle_f@ukw.de (F.M.); fassnacht_m@ukw.de (M.F.); 3 Department of Experimental and Clinical Biomedical Sciences, University of Florence, 50134 Florence, Italy; letizia.canu@unifi.it; 4 Department of Endocrinology, Cochin Hospital, Assistance Publique Hôpitaux de Paris, 75014 Paris, France; rossella.libe@cch.aphp.fr; 5 Endocrinology Unit, Department of Medicine DIMED, University-Hospital of Padua, 35128 Padua, Italy; filippo.ceccato@unipd.it; 6 Department of Endocrinology, Medizinische Klinik und Poliklinik IV, Klinikum der Universität München, LMU München, 80336 Munich, Germany; felix.beuschlein@usz.ch; 7 Klinik für Endokrinologie, Diabetologie und Klinische Ernährung, Universitätsspital Zürich (USZ), Universität Zürich (UZH), 8091 Zurich, Switzerland; 8 Endocrinology in Charlottenburg, 10627 Berlin, Germany; marcusquinkler@t-online.de; 9 Statistical Unit, Department of Clinical and Biological Sciences, University of Turin, 10043 Turin, Italy; paola.berchialla@unito.it; 10 Département dImagerie, Service dOncologie Endocrinienne, Université Paris-Saclay, 94805 Villejuif, France; eric.baudin@gustaveroussy.fr; 11 Division of Endocrinology, Department of Medicine and Research Center, Centre Hospitalier de l’Université de Montréal (CHUM), Montréal, QC 3840, Canada; isabelle.bourdeau@umontreal.ca (I.B.); andre.lacroix@umontreal.ca (A.L.); 12 Endocrinology, Hospital Niguarda Ca’ Granda, 20121 Milan, Italy; paola.loli@clinicasancarlo.it; 13 Department of Medical and Surgical Specialties, Radiological Sciences, and Public Health, Spedali Civili Hospital, University of Brescia, 25123 Brescia, Italy; alfredo.berruti@gmail.com; 14 Department of Endocrinology, University Hospital Zagreb, 10000 Zagreb, Croatia; darko.kastelan@gmail.com; 15 Department of Internal Medicine Maxima MC, 5631 Eindhoven/Veldhoven, The Netherlands; h.haak@mmc.nl; 16 Division of General Internal Medicine, Department of Internal Medicine, Maastricht University Medical Centre+, 6229 Maastricht, The Netherlands; 17 Comprehensive Cancer Center Mainfranken, University of Würzburg, 97080 Würzburg, Germany

**Keywords:** mitotane, adjuvant treatment, adrenocortical cancer, recurrence, recurrence free survival, timing

## Abstract

A relevant issue on the treatment of adrenocortical carcinoma (ACC) concerns the optimal duration of adjuvant mitotane treatment. We tried to address this question, assessing whether a correlation exists between the duration of adjuvant mitotane treatment and recurrence-free survival (RFS) of patients with ACC. We conducted a multicenter retrospective analysis on 154 ACC patients treated for ≥12 months with adjuvant mitotane after radical surgery and who were free of disease at the mitotane stop. During a median follow-up of 38 months, 19 patients (12.3%) experienced recurrence. We calculated the RFS after mitotane (RFSAM), from the landmark time-point of mitotane discontinuation, to overcome immortal time bias. We found a wide variability in the duration of adjuvant mitotane treatment among different centers and also among patients cared for at the same center, reflecting heterogeneous practice. We did not find any survival advantage in patients treated for longer than 24 months. Moreover, the relationship between treatment duration and the frequency of ACC recurrence was not linear after stratifying our patients in tertiles of length of adjuvant treatment. In conclusion, the present findings do not support the concept that extending adjuvant mitotane treatment over two years is beneficial for ACC patients with low to moderate risk of recurrence.

## 1. Introduction

Adrenocortical carcinoma (ACC) is a rare tumor characterized by an aggressive disease course that limits long-term survival [1,2,3]. Disease-specific outcomes are better for patients bearing early-stage tumors that can be resected completely; however, post-operative recurrence of ACC may be considered as part of the natural history of the disease [4,5,6,7,8]. We have recently reported a recurrence rate of 62.5% among 152 patients with stage I to III ACC who underwent complete macroscopic resection, with a five-year recurrence-free survival rate of 38.1% [9].

The remarkable propensity of ACC towards recurrence despite complete surgical removal makes a strong case for an adjuvant therapy. Until now, the most followed adjuvant approach relied on mitotane, an old adrenolytic drug specifically approved for treatment of advanced ACC [10,11]. Use of adjuvant mitotane increased in clinical practice following the observation that adjuvant mitotane treatment was associated with prolonged recurrence-free survival (RFS) compared to surveillance without active treatment after surgery, in a retrospective study of 177 ACC patients managed at different institutions using either adjuvant mitotane or no treatment. In this cohort study, we included 47 patients followed at Italian reference centers that systematically adopted adjuvant mitotane to all radically operated ACC, and a group of 55 Italian patients and 75 German patients followed in centers not giving any post-operative treatment [12]. In this study, we showed that adjuvant mitotane treatment was associated with a significant survival advantage. Despite the retrospective nature of the study, this finding informed clinical practice, although adjuvant mitotane is not universally accepted and some experts argue against the value of this approach [13,14]. Critics of adjuvant mitotane therapy evoke the drug-related toxicity, the complexity of caring for patients on treatment, and the long duration of a treatment course [15]. 

The overall level of evidence available on adjuvant mitotane can be graded as low, and all recommendations are based on retrospective, non-randomized studies, plagued by potential bias and confounding [10,16]. Despite this evidence gap, adjuvant mitotane is advised for patients with ACC at high risk of recurrence in the clinical guidelines endorsed by the European Society for Endocrinology (ESE)–European Network for the Study of Adrenal Tumours (ENSAT) and by the European Society for Medical Oncology (ESMO) [10,17]. The guidelines underline that scant information is currently available on many aspects dealing with practical management of mitotane therapy. Recommendations on how to conduct adjuvant mitotane treatment are mostly based on expert opinions stemming from personal experience and practice [18]. As a consequence, care of patients treated with mitotane is heterogeneous depending on local preferences.

One of the most relevant and uncertain issues concerns the optimal duration of adjuvant mitotane treatment. The ESE-ENSAT guidelines and ESMO guidelines suggest continued use of adjuvant mitotane for at least 2 years, but not longer than 5 years [10,17]. Since no study has ever specifically addressed this issue, this recommendation is based on the observation that most recurrences of ACC occur within two years after resection, while after 5 years, the rate of recurrence is too low to justify continuation of adjuvant therapy. 

What is the optimal duration of adjuvant mitotane treatment, however, remains controversial, and practice varies even among referral centers. This issue has important consequences on both patient-centered outcomes, due to the unwanted effects of treatment and their impact on quality of life and health-care organizations, as surveillance of patients on mitotane is demanding and resource-consuming.

We tried to answer this question by organizing an international, multicentric, retrospective study aimed at assessing whether a correlation does exist between duration of adjuvant mitotane treatment and recurrence-free survival of patients with ACC. 

## 2. Patients and Methods

### 2.1. Patients

We did an international, multicenter, retrospective analysis on 154 patients with ACC treated with adjuvant mitotane after radical surgery. Thirteen European centers and one center in Canada participated in the study. 

To be included, patients had to meet the following inclusion criteria: age of 18 years or older at the time of diagnosis; histologically confirmed diagnosis of ACC (based on Weiss score [19]; ENSAT stage I-III [20]; R0 or Rx tumor resection, defined on the basis of a surgical report, pathology report, and post-operative imaging); treatment with adjuvant mitotane for at least 12 months following surgery; and clinical status being free of disease at the time of mitotane discontinuation. Exclusion criteria were: residual disease after resection, defined both microscopically or macroscopically (resection status, R1 or R2); patients concomitantly treated with other therapies (e.g., chemotherapy or radiotherapy); or patients experiencing ACC recurrence during adjuvant mitotane therapy. Follow-up for this study was closed in December 2017.

### 2.2. Methods

All data were obtained by reviewing patient history, medical records, and source documents. Data were processed by skilled and experienced personnel using specifically tailored data forms. We reported clinical and demographical characteristics, the date and type of surgery, stage at diagnosis, pathology reports (Weiss score and Ki-67 index), hormonal status, date of start and stop of mitotane treatment and reason for stops, date of recurrence and type of recurrence (single or multiple, local or distant), and the date of last follow-up or death. Date of diagnosis was defined as the date of surgery. Tumor stage was established according to the ENSAT classification (I, confined tumors ≤5 cm; II, confined tumors >5 cm; III, positive lymph nodes or infiltrating neighboring organs/veins without distant metastases; IV, distant metastases) [20]. Date of recurrence was defined as the date of radiological evidence of a new lesion. Patients underwent imaging follow-up (abdominal and thoracic computed tomography) every 3–4 months. Modalities of mitotane treatment, such as the initial high- or low-dose regimen, dose titration, and eventual dose changes due to toxicity were done according to local center preferences.

### 2.3. Statistical Analysis

The primary endpoint was to determine whether a correlation between duration of adjuvant mitotane treatment and patient survival did exist. 

Frequencies and percentages were calculated for categorical data, and the median and interquartile range for continuous data. Differences in categorical variables were analyzed by means of the chi-squared test or Fisher test, as appropriate, while differences in continuous variables were analyzed by the Mann–Whitney U test. The survival curves were estimated with the Kaplan–Meyer product limit method. Recurrence-free survival (RFS) was calculated from the time of initial surgery to the first radiological evidence of recurrence. To adjust for the immortal bias due to the selection of patients who did not have recurrence on active treatment, we calculated the recurrence-free survival rate after adjuvant mitotane discontinuation (RFSAM) from the time of discontinuation of mitotane to ACC recurrence or end of follow-up. We calculated the overall survival rate after adjuvant mitotane discontinuation (OSAM) from the time of discontinuation of mitotane to the date of death. Patients who did not experience either of those events (recurrence or death) were censored at the date of the last follow-up visit for the specific survival analysis. Cox proportional hazards regression models were fitted to determine prognostic factors on survival. The following potential predictive factors for RFS and RFSAM were investigated: patient sex and age, tumor stage, hormone secretion, Weiss score, Ki67 index, mitoses, resection status, and duration of mitotane treatment. A genetic algorithm was employed to select the variables that resulted in the best-fitted model according to AIC score [21]. Firth correction was applied to reduce the bias due to the small number of events [22]. All reported P values are two-sided. P-values of less than 0.05 were considered as statistically significant. All statistical analyses were performed using R version 4.0.2.

## 3. Results

Baseline characteristics of patients are reported in Table 1.

In our series, female sex was more prevalent, and ACC presented, in most cases, as a stage II hormone-secreting tumor. The present series was skewed toward low-grade tumors with a Ki67 index of less than 10%. Median duration of adjuvant mitotane therapy was 33 months (IQR, 24–59), and median follow-up after mitotane discontinuation was 38 months (IQR, 24–61).

We stratified our patients into three groups by treatment duration (expressed in tertiles); group 1 included patients treated for 13–25 months, group 2 for 26–48 months, and group 3 for 49–143 months, respectively. Group 3 had a higher Ki67 index, longer RFS compared to groups 1 and 2, and longer RFSAM compared to group 2 (Table 2).

In most cases, mitotane was interrupted at the end of the scheduled period. Only in a few patients, treatment-related unwanted effects induced mitotane stop (Table 3), mainly during the second year of therapy.

**Figure 1:**
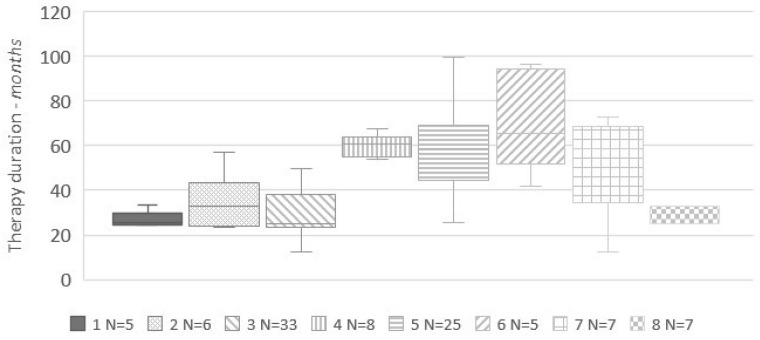
Distribution of the duration of adjuvant mitotane for each center (only centers with at least five patients have been included in this analysis). 1 = Berlin; 2 = Munich; 3 = Wurzburg; 4 = Florence; 5 = Orbassano; 6 = Padua; 7 = IGR (Villejuif); 8 = Montreal. N = number of patients.

After excluding patients in which mitotane withdrawal was determined by adverse effects, we observed a wide variability in the duration of adjuvant mitotane, either among different centers or in the same center, as shown in Figure 1.

In our series, 19 patients experienced recurrence after mitotane discontinuation. Recurrence types were almost equally distributed between those which were local (10 cases, 53%) and distant (9 cases, 47%); the last ones mainly in the lung (6 cases). After mitotane discontinuation, death occurred in three patients, but in only one case the death was cancer-related.

**Figure 2:**
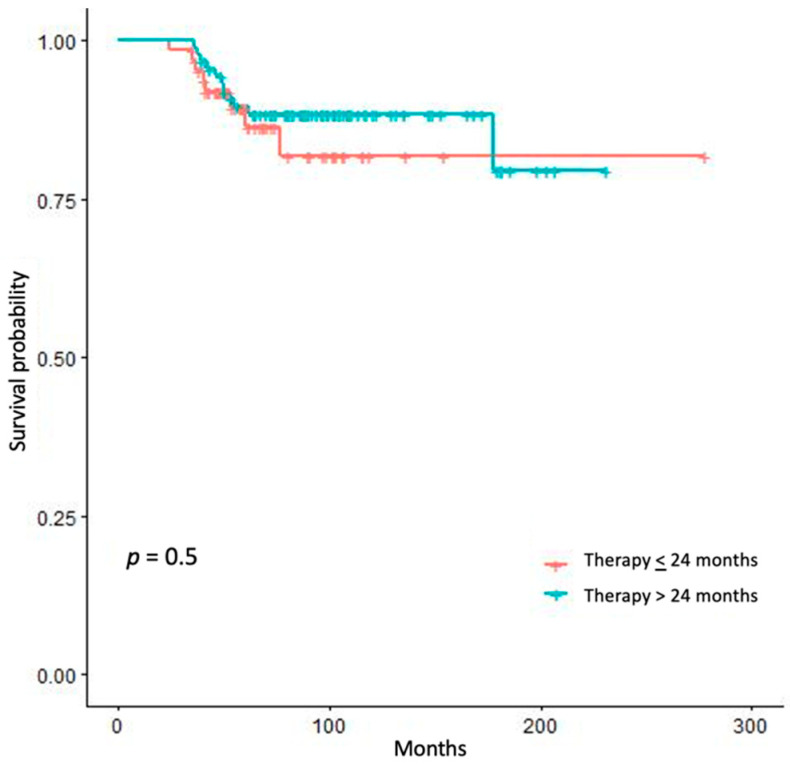
Kaplan–Meier estimates of recurrence-free survival (RFS) in patients treated <24 months versus patients treated >24 months.

**Figure 3:**
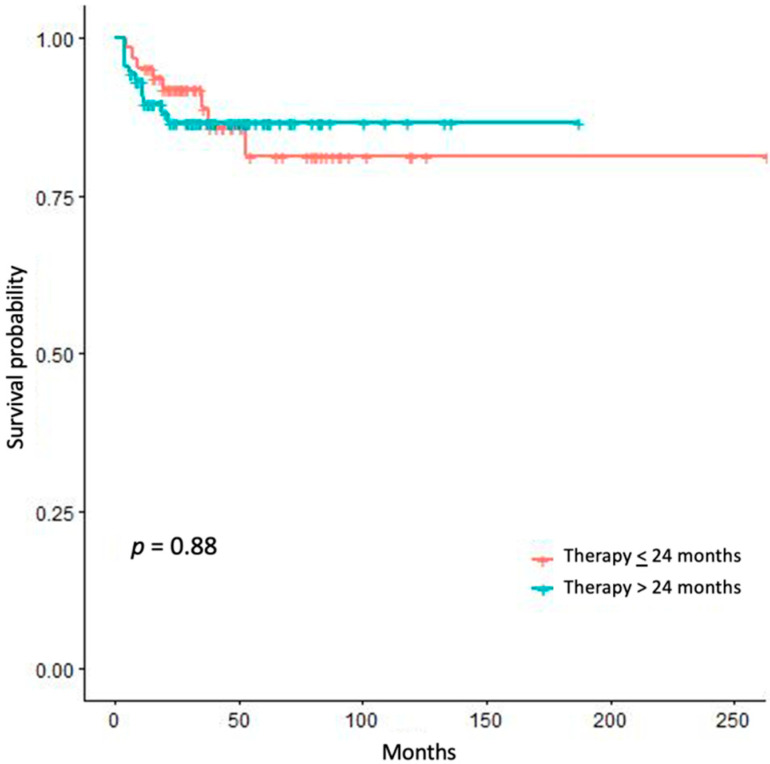
Kaplan–Meier estimates of recurrence free survival after adjuvant mitotane discontinuation (RFSAM) in patients treated <24 months vs. patients treated >24 months.

To assess where any correlation did exist between adjuvant mitotane duration and RSF, we tried different approaches. We stratified our patients by the value of 24 months’ treatment duration. The comparison of the survival curves of patients treated up to 24 months vs. patients treated for a longer period, both for RFS (Figure 2) and RFSAM (Figure 3), did not show any significant difference.

We performed univariate analyses, both for RFS (Table 4) and RFSAM (Table 5), without identifying any significant prognostic factors. 

At a multivariate level, the variables’ duration of adjuvant mitotane, which was modelled with a spline to account for non-linearity, sex, and Weiss were selected in the best-fitted model. Duration of adjuvant mitotane was the only statistically significant factor associated with RFS (HR 0.549, 95% CI 0.306–0.983; *p* = 0.044). The HR was calculated on a difference of 18 months in the duration of therapy, that is, 18 months’ increase of adjuvant mitotane therapy duration is associated with about 45% reduction in the hazard of RFS. No statistically significant factor resulted to be associated with RFSAM, or OSAM.

## 4. Discussion

The present findings do not support the concept that a longer duration of mitotane therapy (more than 2 years) is associated with a survival advantage. Since the present series was enriched with low-risk tumors, these results may be not generalizable to high-risk ACC. Although RFS was prolonged in patients treated for more than 4 years, this finding may likely be the consequence of immortal time bias. 

The inclusion criteria of the study may induce an immortal bias in patients scheduled to be treated for longer periods, since these patients could not have recurred in the months prior to mitotane discontinuation, as otherwise they would have been excluded from analysis. For this reason, we primarily focused on the outcome after the end of scheduled adjuvant treatment, of whatever duration it was. We calculated the recurrence-free survival rate after mitotane (RFSAM), from the landmark time-point of mitotane discontinuation, since landmark analysis is a method used to overcome immortal time bias [23].

The duration of mitotane therapy was a factor associated to RFS but not RFSAM, and this militates against an actual benefit of prolonging adjuvant mitotane treatment. Along these lines, the breakdown of RFS by 24 months of treatment duration, which is the recommended time-length of adjuvant mitotane according to the ESE-ENSAT and ESMO guidelines [10,17], did not disclose any survival advantage of patients treated for longer. Moreover, the relationship between treatment duration and the frequency of ACC recurrence was not linear after stratifying our patients in tertiles of length of adjuvant treatment.

An interesting finding is that a large variability in the duration of adjuvant mitotane treatment does appear between different centers, and also among patients cared for at the same center. This figure reflects uncertainty on management and heterogeneous practice. However, it appears that physicians were more eager to treat patients with unfavorable prognostic factors (higher Ki-67 index) for longer periods, although our series was selected toward low-risk tumors due to the specific inclusion criteria of the study (patients who did recur on treatment were excluded). That said, a sort of “geographical” pattern does appear, since at centers in Germany, adjuvant mitotane was generally discontinued after two years, whereas in Italian centers it was usually more prolonged. In these centers, however, there was a huge variability between patients, suggesting that a tailored approach, taking into account patient preferences and biological characteristics of ACC, was followed. The heterogeneity in practice between expert centers underlines the lack of evidence on this issue since, to the best of our knowledge, this is the first study that specifically addresses the issue of the duration of adjuvant mitotane therapy. As a matter of fact, duration of adjuvant mitotane was very heterogenous between studies on this topic [14,24,25,26,27,28]. 

The main issue with a retrospective analysis of treatment duration is that in many cases, the length of adjuvant treatment is set by the timing of ACC recurrence, and not by the planned treatment schedule. In this retrospective multicenter study carried out in referral centers for ACC that are part of ENSAT, we tried to overcome this problem by including only patients treated with adjuvant mitotane for at least 12 months, in which mitotane was suspended for reasons different from recurrence of disease. Consequently, all patients were free of disease at the end of adjuvant therapy. 

Recognizing that a prospective randomized trial that includes patients with ACC treated with adjuvant mitotane for different, pre-specified time lengths is the best way to define what is the optimal duration of treatment, it can be plainly accepted that such a trial is not on the horizon for the near future. Up to now, only two randomized trials have been concluded on ACC [29,30], and this outlines how it is challenging to implement a randomized trial in a rare tumor such as ACC. Therefore, a well-designed retrospective study is almost all that can be done to answer this important clinical question. 

## 5. Conclusions

In this study, we have addressed the challenging issue of identifying the optimal duration of adjuvant mitotane therapy in a retrospective analysis. We tried to overcome the bias and confounding inherent to a retrospective analysis of treatment duration by multiple approaches. With all the disclosed limits of our study, the present findings do not support the concept that extending adjuvant mitotane treatment over two years is beneficial for patients with ACC at low risk of recurrence. Conversely, patients with ACC at high risk of recurrence were under-represented in this study, thus precluding any definitive recommendations.

Answering the question of what is the optimal duration of adjuvant mitotane is an unmet clinical need, since current practice is heterogeneous and mainly dependent on personal preferences and expertise. To the best of our knowledge, no previous study has specifically addressed this point, and the recommendation of a standard duration of adjuvant mitotane treatment of two years is not based on specific evidence. Lacking randomized studies, which will be hardly feasible in the future, the present study does provide the only evidence available on this complex issue. Finding that no obvious advantage is associated to prolonged adjuvant mitotane treatment provides some guidance for the care of patients with ACC, and sparing low-risk patients from long exposure to a toxic treatment matters for clinical practice. 

## Figures and Tables

**Table 1:** Baseline characteristics of patients.

Characteristics	Valid Cases (*N*)	Values
Sex, N (%)	154	
Male		51 (33%)
Female		103 (67%)
Age at diagnosis, years	154	
Median (IQR)		45 (34–54)
Tumor stage at diagnosis, *N* (%)	154	
Stage I		14 (9%)
Stage II		110 (71%)
Stage III		30 (19%)
Hormone secretion at diagnosis, *N* (%)	113	
No		43 (38%)
Yes		70 (62%)
Glucocorticoid		41 (59%)
Androgen		21 (30%)
Aldosterone		5 (7%)
Other		3 (4%)
Tumor size, cm	148	
Median (IQR)		10 (7–15)
Ki67 at diagnosis	125	
Median (IQR)		10 (5–20)
≤10%		75 (60%)
>10%		50 (40%)
Weiss at diagnosis	132	
Median (IQR)		6 (4–6)
Duration of mitotane therapy, months	154	
Median (IQR)		33 (24–59)

IQR = interquartile range.

**Table 2:** Baseline characteristics of patients in different groups stratified by duration of therapy.

Characteristics	Group 1 n. 52 Patients (Treated for 13–25 Months)	Group 2 n. 51 Patients (Treated for 26–48 Months)	Group 3 n. 51 Patients (Treated for 49–143 Months)	*p*-Value
Sex, *N* (%)				0.15
Male	16 (31%)	13 (25%)	22 (43%)	
Female	36 (69%)	38 (75%)	29 (57%)	
Age at diagnosis, years				0.15
Median (IQR)	47.5 (38.5–58)	45 (32.5–53)	43 (34–51.5)	
Tumor stage at diagnosis, *N* (%)				0.44
Stage I	4 (8%)	5 (10%)	5 (10%)	
Stage II	41 (79%)	37 (72%)	32 (63%)	
Stage III	7 (13%)	9 (18%)	14 (27%)	
Hormone secretion at diagnosis, *N* (%)				0.41
No	31 (60%)	24 (47%)	29 (57%)	
Yes	21 (40%)	27 (53%)	22 (43%)	
Size tumor at diagnosis, cm				0.80
Median (IQR)	9.5 (7.2–14.5)	10.5 (7.6–14)	10 (6.7–15.5)	
Ki67 at diagnosis				0.014
Median (IQR)	10 (5–10)	10 (5–19)	15 (6–23)	
Weiss at diagnosis				0.39
Median (range)	5 (4–6)	6 (4–7)	6 (5–7)	
Recurrence, *N* (%)				**0.001**
No	47 (90%)	38 (75%)	50 (98%)	
Yes	5 (10%)	13 (25%)	1 (2%)	
RFS, months				**<0.001**
Median (IQR)	61 (49–97)	59 (48–85)	108 (90–151)	
RFSAM, months				**0.002**
Median (IQR)	38 (26–78)	22 (11–47)	35 (24–62)	
OSAM, months				0.19
Median (IQR)	44 (26–78)	37 (22–53)	35 (27–62)	

IQR = interquartile range; RFS = recurrence free survival; OSAM = overall survival after adjuvant mitotane discontinuation; RFSAM = recurrence free survival after adjuvant mitotane discontinuation. Statistically significant differences are presented in bold.

**Table 3:** Causes of mitotane discontinuation in different groups stratified by duration of therapy.

Causes of Mitotane Discontinuation	Group 1 n. 52 Patients (Treated for 13–25 Months)	Group 2 n. 51 Patients (Treated for 26–48 Months)	Group 3 n. 51 Patients (Treated for 49–143 Months)
End of schedule	30 (57.7%)	38 (74.5%)	47 (92.2%)
Adverse effects	20 (38.5%)	8 (15.7%)	2 (3.9%)
Unattainable target level	0	2 (3.9%)	0
Severe concomitant disease	1 (1.9%)	1 (1.9%)	0
Other *	1 (1.9%)	2 (3.9%)	2 (3.9%)

* patient willing, unexpected pregnancy.

**Table 4:** Univariate analysis of predictive factors for recurrence-free survival (RFS).

Univariate Analysis	Diff	HR	95% CI		*p*
Duration of mitotane therapy ^+^		1.302	0.509	3.334	0.58
_§_ R status		0.722	0.208	2.503	0.61
^‡^ * Hormone secretion		1.441	0.571	3.640	0.44
* ° Stage		0.917	0.272	2.526	0.87
* Tumor size	7.925	0.942	0.514	1.727	0.85
* Weiss	2.000	1.589	0.861	2.932	0.14
* Ki67%	15.000	0.805	0.426	1.521	0.50

* at diagnosis; Reference categories: ^+^ patients treated with mitotane ≤27 months, ^‡^ Secreting tumors, ° Stage III, ^§^ RX.

**Table 5:** Univariate analysis of predictive factors for recurrence free survival after adjuvant mitotane discontinuation (RFSAM).

Univariate Analysis	Diff	HR	95% CI		*p*
Duration of mitotane therapy ^+^		0.894	0.354	2.257	0.812
^§^ R status		0.843	0.243	2.924	0.788
^‡^ * Hormone secretion		1.357	0.543	3.391	0.513
* ° Stage		1.118	0.342	2.993	0.838
* Tumor size	7.925	0.977	0.532	1.792	0.939
* Weiss	2.000	1.766	0.957	3.260	0.069
* Ki67%	15.000	0.820	0.442	1.521	0.529

* at diagnosis; Reference categories: ^+^ patients treated with mitotane ≤27 months, ^‡^ Secreting tumors, ° Stage III, ^§^ RX.

## Data Availability

The data presented in this study are available on request from the corresponding author.
